# Sutureless repair for vena cava bleeding using an elastomeric sealant in a porcine model

**DOI:** 10.1093/icvts/ivad122

**Published:** 2023-07-24

**Authors:** Yoshinori Nakahara, Retsu Tateishi, Shunya Ono, Takeyuki Kanemura

**Affiliations:** Department of Cardiovascular Surgery, IMS Katsushika Heart Centre, Tokyo, Japan; Department of Cardiovascular Surgery, IMS Katsushika Heart Centre, Tokyo, Japan; Department of Cardiovascular Surgery, IMS Katsushika Heart Centre, Tokyo, Japan; Department of Cardiovascular Surgery, IMS Katsushika Heart Centre, Tokyo, Japan

**Keywords:** Venous bleeding, Haemostatic agent, Sutureless repair technique

## Abstract

To address the issue of bleeding from the vena cava, a common complication of cardiac and aortic surgeries, we developed a sutureless repair technique using Hydrofit, an elastomeric sealant. In a porcine model, we surgically incised the inferior vena cava and applied the elastomeric sealant with a pericardial patch, achieving complete haemostasis through manual compression. This simple and rapid technique demonstrates effective sutureless haemostasis for the vena cava.

## INTRODUCTION

Bleeding complications arising from the vena cava can occur during major cardiovascular surgical procedures. Although major venous injuries sustained during general surgery are relatively rare, these injuries can result in serious complications, including increased perioperative morbidity and mortality, without the aid of cardiopulmonary bypass [1]. Haemostasis may pose a challenge in the absence of cardiopulmonary bypass, in deep or narrow surgical fields, or when the field of vision is limited. Therefore, in this study, we investigated the feasibility of sutureless repair techniques in a porcine model.

## CASE REPORT

Animal experiments were conducted ethically, following approved guidelines. Approval was obtained from the Ethical Committee of Katsushika Heart Centre (reference number: 20230128-1).

**Figure 1:**
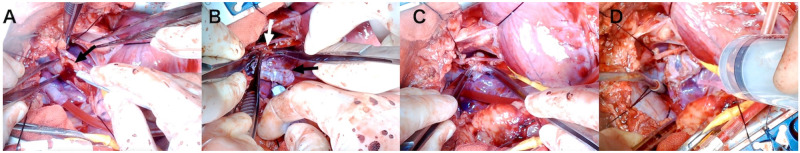
Surgical scheme. (**A**) Incision was made in the inferior vena cava to create a haemorrhage model (black arrow). (**B**) Hydrofit was applied to the injury site (white arrows) and pericardium (black arrows). (**C**) The patch was attached to the inferior vena cava injury site, and 2-min manual compression was performed. (**D**) Complete haemostasis was achieved.

This study employed a porcine model under general anaesthesia. Median sternotomy was performed, followed by systemic heparinization. Cardiopulmonary bypass was initiated by accessing the ascending aorta and the right atrium. After the successful implementation of extracorporeal circulation, decannulation from the cardiopulmonary bypass was carried out. While under heparinization, incisions measuring 2 cm were made in the inferior vena cava using a scalpel to create a haemorrhage model (Fig. 1A and Video 1). Hydrofit (Sanyo Chemical Industries, Kyoto, Japan), an elastomeric sealant that reacts with water present on the surface of the living tissues, along with a pericardial patch was applied to the pericardium and placed over the bleeding area (Fig. 1B and C). A minor bleeding was observed at the incision site, along with a moist surgical field when Hydrofit was applied to the vena cava. Nonetheless, complete haemostasis was achieved after performing manual compressions for 2 min (Fig. 1D).

## DISCUSSION

Using Hydrofit, an elastomeric sealant, effectively stops vena cava bleeding without sutures. It offers a novel, easy-to-use method completed quickly. The sealant, composed of reactive isocyanate groups and a fluorine-containing polyether polyurethane prepolymer, rapidly reacts with water on the tissues to create a strong adhesive film [2]. Unlike fibrin glue or TacoSeal, Hydrofit works well in moist environments. Furthermore, the viscosity of the sealant allows precise application without spreading or flowing into the vessels. Lastly, Hydrofit also can work in cases of coagulopathy, such as disseminated intravascular coagulopathy, as it is not related to the blood coagulation cascade.

Hydrofit reportedly exhibited remarkable haemostatic effects in various studies. One study pertained to left ventricular rupture [3], whereas the other focused on ventricular septal rupture [4]. These studies reported that Hydrofit achieved complete left ventricular haemostasis. Therefore, it appears that the adhesive strength of Hydrofit is sufficient to control venous bleeding, which occurs under low venous pressure (5–15 mmHg). However, the long-term safety and durability for use of venous haemostasis remains uncertain, and the absence of postoperative follow-up or histopathological examination constituted a limitation in our study. We present 2 arguments supporting the durability of this haemostatic technique in the vena cava. First, in our case, we combined Hydrofit with a pericardium patch. Even if Hydrofit loses haemostatic effect, pericardium filling prevents further bleeding. Second, venous bleeding complications have rarely been documented during remote phase. We previously reported a case of coronary sinus rupture, which was arrested using Hydrofit [5]. In this case, haemostasis was achieved using the pericardium, and the patient has been free of any complications for 2 years. Together, this suggests that durability of Hydrofit for venous haemostasis is not a concern.

Sutureless repair arrested bleeding from a significant source of haemorrhage in the vena cava, suggesting that it may be highly effective for managing venous oozing and bleeding during other surgical procedures also. Further evaluation of its long-term durability and efficacy in human beings is required.

## Data Availability

The data underlying this article will be shared on reasonable requests to the corresponding author.
